# GENDER DIFFERENCES IN SLEEP QUALITY, FATIGUE, AND DEPRESSIVE SYMPTOMS AMONG ADULTS OVER 50 WITH TOTAL BLINDNESS

**DOI:** 10.1093/geroni/igad104.0684

**Published:** 2023-12-21

**Authors:** Soyoung Choi, Tracie Harrison

**Affiliations:** University of Illinois at Urbana-Champaign, Urbana, Illinois, United States; The University of Arkansas for Medical Sciences, Little Rock, Arkansas, United States

## Abstract

Visual limitations and blindness have been associated with poor sleep quality, increased fatigue, and a higher risk of depressive symptoms in the aging population. In this study, we aimed to investigate sleep quality, fatigue, and depressive symptoms in US adults over the age of 50 with total blindness, and to examine potential gender differences. The study included 88 participants (59 females, mean age 61.34±8.04; 19 males, mean age 62.32±8.17) who completed an online survey providing data on socioeconomic status, comorbidities, sleep quality, fatigue, and depressive symptoms. Descriptive and bivariate analyses were performed using R software and its required packages. While females slept less than males (t = -2.98, p <.05), there was no significant difference in sleep quality (t = 1.82, p = .08) or depressive symptoms (t = 0.51, p = 0.62). Fatigue levels were significantly higher in females than males (t = 2.53, p <.05). Poorer sleep quality was associated with increased fatigue and severe depressive symptoms in both subgroups. The findings indicate potential gender effects, however, given the small sample size and limitations of the cross-sectional design, further longitudinal research is needed to fully understand gender differences in sleep quality, fatigue, and depressive symptoms among adults over 50 with visual limitations.

